# Role of the Vibriolysin VemA Secreted by the Emergent Pathogen *Vibrio europaeus* in the Colonization of Manila Clam Mucus

**DOI:** 10.3390/microorganisms10122475

**Published:** 2022-12-15

**Authors:** Clara Martinez, Sergio Rodriguez, Ana Vences, Juan L. Barja, Alicia E. Toranzo, Javier Dubert

**Affiliations:** Department of Microbiology and Parasitology, Aquaculture Institute & CIBUS-Faculty of Biology, University of Santiago de Compostela, 15782 Santiago de Compostela, Spain

**Keywords:** vibriosis, *Vibrio europaeus*, M4 zinc-metalloprotease, vibriolysin, VemA, shellfish aquaculture, Manila clam, bivalves, mucus

## Abstract

*Vibrio europaeus* is an emergent pathogen affecting clams, oysters and scallops produced in the most important countries for bivalve aquaculture. Studies concerning virulence factors involved in the virulence of *V. europaeus* are very scarce despite its global significance for aquaculture. Zinc-metalloproteases have been described as a major virulence factor in some *Vibrio* spp., although their contribution and role in the virulence of *V. europaeus* is not clear. To address this, we have studied an extracellular zinc-metalloprotease (VemA) encoded by *V. europaeus*, which was identified as a vibriolysin, highly conserved in this species and homologous in other pathogenic and non-pathogenic species. Virulence challenge experiments demonstrated that infection processes were faster when Manila clam larvae and juveniles were infected with the wildtype rather than with a mutant defective in the *vemA* gene (Δ*vemA*). *V. europaeus* was able to resist the bactericidal action of mucus and displayed a chemotaxis ability favoured by VemA to colonize the body mucus of clams and form a biofilm. The overall results suggest that VemA, although it is not a major virulence factor, plays a role in the colonization of the Manila clam mucus, and thus boosts the infection process as we observed in virulence challenge experiments.

## 1. Introduction

Molluscan aquaculture is the second most important activity within the world aquaculture [1]. Manila clams (*Ruditapes philliphinarum*) and Pacific oysters (*Crassostrea gigas*) are the most important bivalve species in aquaculture, accounting the 8% of the global aquaculture production [1]. As molluscan filter feeders, they filter large quantities of water containing microalgae, bacteria and detritus from the environment in which they live [2,3]. Thus, they have the ability to bioaccumulate high bacterial concentrations in their tissues, including bacterial pathogens [3,4,5]. To fight the pathogens, bivalves rely primarily on (i) the pallial cavity fluid and its associated mucus, which has important functions such as lubrication, particle capture and antimicrobial activity being the first line of defence against microorganisms [2]; and (ii) both cellular and humoral defence factors in the haemolymph [2,3].

Bacterial pathogenic species belonging to the genus *Vibrio* are the most important threat to bivalves, constraining the expansion of bivalve aquaculture [4]. The first reports of *Vibrio* disease in bivalves (known as “vibriosis”) were published more than 50 years ago; however, this global problem is still unsolved [4]. Among the pathogenic species, *Vibrio europaeus* represents an excellent bacterial model to study the vibriosis in bivalves due to: (i) the negative impact on the worldwide aquaculture: it is responsible of recurrent mass mortalities in the world’s most important bivalve producers, such as France, Spain, Chile and the US [6,7,8,9,10,11], and (ii) its wide range of hosts affecting different bivalve species and stages of the bivalve’s life cycle: *V. europaeus* infects a wide range of different clam, oyster and scallop species, including Manila clam and Pacific oyster, and even at different stages of development such as larvae, spat and juveniles [6,7,8,9,10,11,12,13,14,15,16]. Phylogenetically, this taxon was initially described as a subspecies of *V. tubiashii*, formed by two pathogenic subspecies with significance for shellfish aquaculture: *V. tubiashii* subsp. *tubiashii* and *V. tubiashii* subsp. *europaeus* [9]. However, this taxon was later reclassified and elevated to the rank of species supported by a novel phylogenetic analysis based on genome-to-genome comparisons and chemotaxonomic and phenotypic differences [14].

Virulence is a multifactorial trait that results from the sequential action of several microbial factors that enable colonization, proliferation and evasion of the host immune system and ultimately causes disease [17]. Regarding to the virulence factors, Spinard et al. [18] found in silico different proteases encoded by the *V. europaeus* CECT 8136 genome, including two metalloproteases, three putative haemolysins and phospholipases. Mersni-Achour et al. [12] demonstrated that the extracellular products (ECPs) of the strain *V. europaeus* 07/118 T2 inhibited the adhesive capacity and phagocytic activity of *C. gigas* haemocytes. Complementary biochemical analyses showed that the proteolytic fraction of ECPs contained an active and thermostable extracellular zinc-metalloprotease(s). Later, Mersni-Achour et al. [13] fractioned the *V. europaeus* ECPs to study the toxicity of the two major fractions (F1 and F2). They found that F1 was less toxic (43% mortality) in contrast to F2, which it was responsible of the 70% mortality in Pacific oyster larvae. Interestingly, F2 contained a unique protein identified as an extracellular zinc-metalloprotease, hereinafter designated as VemA (*V. europaeus* metalloprotease A). Other extracellular zinc-metalloproteases have been also reported as a virulence factor required for the full virulence in other *Vibrio* pathogens for bivalves such as *V. neptunius* (VnpA), *V. aestuarianus* (Vam) or *V. coralliilyticus* (VcpA and VtpA) [19,20,21,22]. However, the role of the zinc-metalloproteases as a major (or secondary) virulence factor can be different depending on if ECPs or live cells are used. For instance, Le Roux et al. [23] demonstrated that the zinc-metalloprotease Vsm secreted by *V. splendidus* is essential for ECPs toxicity in Pacific oysters; however, it is not necessary when a live mutant (Δ*vsm*) is injected in oysters resulting in high-mortality rates similar to the wildtype. Therefore, to elucidate the role of VemA it is essential to perform in vivo challenge experiments using mutants defective in the *vemA* gene to mimic the natural infection process. To address this, the aims of this work were: (i) to perform in silico analyses to evaluate the presence of VemA in all *V. europaeus* strains (n = 38 strains/genomes); (ii) to construct mutants defective in the *vemA* gene (Δ*vemA*); (iii) to evaluate the contribution of VemA in the virulence of *V. europaeus* by comparison between the wildtype and the Δ*vemA* mutant challenging Manila clam juveniles and larvae; and (iv) to elucidate the role of VemA in chemotaxis, biofilm formation and bacterial proliferation in Manila clam mucus.

## 2. Materials and Methods

### 2.1. Bacterial Strains, Plasmids and Media

The bacterial strains and plasmids used or derived from this study are included in Table 1. *V. europaeus* CECT 8136 was grown on Trypto-Casein Soy agar or broth supplemented with 2% sodium chloride (*w*/*v*) (TSA–2/TSB–2, Condalab, Madrid, Spain) at 25 °C for 24 h. *E. coli* was grown in Luria–Bertani broth supplemented with 1% sodium chloride (*w*/*v*) (LB-1, Condalab, Madrid, Spain) at 37 °C for 24 h. Diaminopimelic acid and thymidine were added at a final concentration of 0.3 mM (*w*/*v*) for auxotrophic strains *E. coli* β3914 and *E. coli* Π3813, respectively (Table 1). Antibiotics were used at the following concentrations: chloramphenicol (Cm; 25 μg mL^−1^ for *E. coli* strains and 5 μg mL^−1^ for *V. europaeus*), erythromycin (Ery; 200 μg mL^−1^) and kanamycin (Kn; 50 μg mL^−1^). A suicide vector for allele exchange pSW7848T [24 carried the *ccdB* gene as a counterselection marker, which is under control of the arabinose promotor (P_BAD_) and induced/repressed by the addition of 0.2% (*w*/*v*) L-arabinose or 1% (*w*/*v*) D-glucose to the growth media.

### 2.2. Search of VemA Homologous and Phylogenetic Analysis

VemA metalloprotease was encoded by the gene *vemA* (WP_069668927) from the *V. europaeus* CECT 8136 (=PP–638) genome [18]. The presence of VemA was verified by BLASTp (i) at the intraspecific level in all *V. europaeus* strains available to the date from the NCBI database (n = 38 strains/genomes) (Table S1); (ii) and at the interspecific level by searching for homologs from NCBI using the non-redundant protein sequences database. The VemA domain prediction was carried out with the UniProt (https://www.uniprot.org) and InterPro servers (https://www.ebi.ac.uk/interpro/) (accessed on 22 Septembre 2022). The resulting heatmaps were performed with R package ComplexHeatmap (v2.11.2) [26]. A phylogenetic tree based on homologous protein sequences was constructed using MEGA11 [27] after a multiple alignment of data by CLUSTAL W [28]. Distances and clustering with the neighbour-joining (NJ) algorithm were determined using bootstrap values based on 1000 replications.

### 2.3. Construction of ΔvemA Mutants by Double Allelic Exchange

An in-frame deletion of the *vemA* gene was performed by double allelic exchange using the pSW7848T suicide plasmid [25]. Upstream and downstream fragments (692 bp) flanking the target gene were amplified by PCR using primers described in Table S2 and Q5 High-Fidelity DNA Polymerase (NEB, Ipswich, MA, USA). The pSW7848T backbone was amplified in a third PCR (Table S2) and the resulting PCR product was digested with DpnI (2 h at 37 °C; NEB, Ipswich, MA, USA) to inactivate any plasmid template. All PCR products were verified by gel electrophoresis and subsequently purified by the QIAquick PCR Purification Kit (QIAGEN, Hilden, Germany) before Gibson assembly reaction using the NEBuilder HiFi DNA Assembly (NEB, Ipswich, MA, USA). Gibson reaction was desalted by dialysis (0.0025 μM filter; Millipore, Burlington, MA, USA), electroporated into *E. coli* Π3813 (strain used for cloning) and the recombinant plasmid (pPC1; Figure S1) verified by (i) colony PCR (Table S2), (ii) restriction enzyme digestion (NdeI; NEB, Ipswich, MA, USA) (iii) and Sanger sequencing (Table S2). Plasmid pPC1 was electroporated into *E. coli* β3914 (donor) for conjugation adapting the protocol described by Hussain et al. [29] to *V. europaeus* (recipient). Overnight cultures were grown as described above and a drop mating assay was adjusted to a 1:3 ratio (donor:recipient) and mating media (TSA–1 plate supplemented with diaminopimelic acid) was incubated at 25 °C for 24 h. Gene deletion involves two homologous recombination events: (i) the first recombination leads to the integration of the recombinant plasmid into the host chromosome by a first homologous recombination (single crossover). For this, insertional mutants were selected from TSA-2 plates supplemented with Cm and glucose 1% (*w*/*v*) and verified by colony PCR (Table S2) and (ii) the pSW7848T backbone is excised from the chromosome through a rare second homologous recombination event (double crossover). Thus, colonies were grown in liquid media to the late logarithmic phase and spread onto TSA-2 plates supplemented with 0.2% arabinose to induce the *ccdB* toxin (Figure S1) under the control of P_bad_. After the second homologous recombination, mutants with a 92% deletion of the *vemA* gene were obtained and verified by (i) colony PCR (Table S2) and (ii) Sanger sequencing (Table S2).

### 2.4. Virulence Challenges

Pathogenicity assays were carried out with Manila clam larvae (180 μm) and juveniles (13 ± 1 mm). *V. europaeus* CECT 8136, PC1-11 and *V. breoganii* C5.5 (used as a negative control) (Table 1) were grown overnight as described above. Bacterial suspensions were made in sterile sea water (SSW) and adjusted to OD_600_ = 1 (~10^8^ CFU mL^−1^). Bacterial concentrations, expressed in colony-forming units per millilitre (CFU mL^−1^), were confirmed by decimal dilution series onto TSA-2 plates. For larvae, virulence assays were performed following Dubert et al. [30] adjusting the bacterial concentrations to 10^4^ CFU mL^−1^ from the initial bacterial suspension and mortalities were evaluated every 24 h. For juveniles, challenges included two steps: (i) infection: tanks were filled with filtered seawater (FSW; 0.22 μM Nalgene Rapid-Flow, Thermo Fisher Scientific, Waltham, MA, USA) containing a bacterial suspension adjusted to a final concentration of 10^7^ CFU mL^−1^. Subsequently, 15 juveniles were added to each tank and kept for 24 h at room temperature (RT = ~20 °C) to actively filtrate the bacteria; and (ii) post-infection: challenged juveniles were taken out the infection tanks for 8 h at RT to internalize the bacteria within the pallial cavity. After this, juveniles were transferred to fresh tanks filled with 200 mL FSW and maintained at RT with aeration. Mortalities were monitored at 8, 20, 32, 44, 56, 68 and 80 h post-infection and clams were immediately removed when the valves were open (dead juveniles), or siphons were not retracted following stimulation (moribund juveniles) and expressed as a percentage of survival. FSW was renewed once per day (or if it was turbid).

Extracellular products (ECPs) and SDS-polyacrylamide gel electrophoresis. *V. europaeus* CECT 8136 and PC1-11 ECPs were collected from overnight cultures in TSB-2. Overnight cultures were centrifuged (6000× *g* for 20 min at 4 °C), cell pellets discarded, and the supernatant collected and filtered through a 0.22 μM filter (Sartorius, Goettingen, Germany). Proteins were precipitated as described by Terceti et al. [31] with 10% (*w*/*v*) trichloroacetic acid (TCA) for 30 min on ice and recovered by centrifugation. Protein pellets were washed in TCA followed by washing in acetone and air-dried. Precipitated proteins were solubilized in SDS-sample buffer (50 mM Tris–HCl, pH 8.8; 2% SDS; 0.05% bromophenol blue; 10% glycerol; 2 mM EDTA, and 100 mM DTT) and subjected to SDS–PAGE in 10 or 12% polyacrylamide gels using the Laemmli discontinuous buffer system stained with Coomassie Brilliant Blue.

### 2.5. Mucus Extraction

Mucus was aspirated from the body surface (mantle and branchia) of non-anaesthetised Manila clam adults (n = 61) using a disposable Pasteur pipette. Mucus was sonicated by two cycles of 30 s at low intensity, centrifuged at 10,000× *g* for 15 min at 4 °C and filtered through a 0.45 μM filter (Millipore, Burlington, MA, USA) to remove debris. Protein concentration was measured using a Pierce BCA Protein assays kit (Thermo Fisher Scientific, Waltham, MA, USA) and mucus was collected and stored at −80 °C until use.

### 2.6. Evaluation of the Bactericidal Activity of the Surface Mucus on Solid Media

*V. europaeus* CECT 8136 and PC1-11 were grown on TSA-2, resuspended in phosphate-buffered saline (PBS, pH 7.4) and spread onto Müeller–Hinton Agar (Oxoid, Hampshire, UK) supplemented with 1% sodium chloride (*w*/*v*) (MHA-1) to a final concentration of 10^5^ CFU mL^−1^. Sterile 6 mm discs impregnated with mucus (20 μL) were applied to the agar plates and incubated at 25 °C for 24 h.

### 2.7. Adherence to Mucus and Biofilm Formation on Polystyrene

Mucus was diluted 1:5 in sterile distilled water and a volume of 100 μL was added to each well of 96-well ELISA polystyrene plates (Jet Biofil, Guangzhou, China). Plates were dried in a laminar flow cabinet for 48 h and subsequently filled with 200 μL of TSB-2 per well. *V. europaeus* CECT 8136 and PC1-11 overnight cultures were diluted 1:100 and incubated at 25 °C for 24 h. Bacterial cells were fixed with methanol (99.8%) stained with 0.5% crystal violet solution (*v*/*v*), washed three times with distilled water and the bound dye was eluted with acetic acid (33%, *v*/*v*) and measured by optical density at 570 nm in a spectrometer (Biotek, Winooski, VT, USA). Results are means calculated from eight replicates.

### 2.8. Chemotaxis Assays

Chemotaxis was evaluated by using the capillary assay described by Valiente et al. [32] with slight modifications. Overnight *V. europaeus* CECT 8136 and PC1-11 cultures were diluted 1:100 and exponentially grown (OD_600_ = 0.9; ~10^7^ CFU mL^−1^) to maximize the number of motile cells before proceeding to the assay. Serial decimal dilutions of the bacterial suspensions were spread onto TSA-2 and incubated as described above. Bacterial cultures were harvested by centrifugation (5000× *g* for 5 min at 4 °C), washed three times in chemotaxis buffer (PBS, 0.01 mM EDTA) and 400 μL dispensed per tube (assays were performed in triplicate). A 50 μL capillary tube (Drummond Scientific, Broomall, PA, USA), sealed at one end and containing the mucus or chemotaxis buffer was inserted in a glass-tube and incubated at 25 °C with the bacterial suspension for 30 min and 1 h. After incubation, the capillaries were removed and externally rinsed with ethanol. Then, the capillary contents were expelled in PBS, 10-fold dilutions prepared and spread on TSA-2 plates. The data shown are means of three independent replicates and the chemotactic responses are expressed as the ratio between the numbers of bacteria in mucus capillaries and the numbers of bacterial in control capillaries only in presence of chemotaxis buffer.

### 2.9. Bacterial Survival and Growth with Mucus

*V. europaeus* CECT 8136 and PC1-11 were grown overnight on TSA-2 and resuspended in SSW to an OD_600_ = 1. A volume of 1 mL adjusted to ~10^5^ CFU mL^−1^ was added into a glass tube with 100 μL of mucus and incubated at 25 °C for 30 min and 24 h. After incubation, samples from each tube were diluted 10-fold and spread on TSA-2 plates. The data shown are means of two independent replicates and the bacterial proliferation is expressed as the ratio between the number of bacteria in mucus after incubation (30 min and 24 h) and the number of bacteria at the beginning of the experiment (time 0).

### 2.10. Statistical Analyses

SPSS (v27; IBM SPSS) was used for statistical analyses. In all cases Kolmogorov–Smirnov tests were previously applied to the normality, and thus, to choose the optimal statistical test applied in each case: (i) for virulence challenges, the statistical significance of differences in percentage survival was determined using the Kruskal–Wallis test; (ii) Student’s *t*-test was used for chemotaxis response, biofilm formation and bacterial proliferation on mucus. *p* values were considered significant when *p* was <0.05 using the Bonferroni-adjusted *p* value.

## 3. Results

### 3.1. VemA Is a Vibriolysin-like Protein, Highly Conserved in V. europaeus with Homologues in Other Pathogenic Vibrio Species

**Figure 1:**
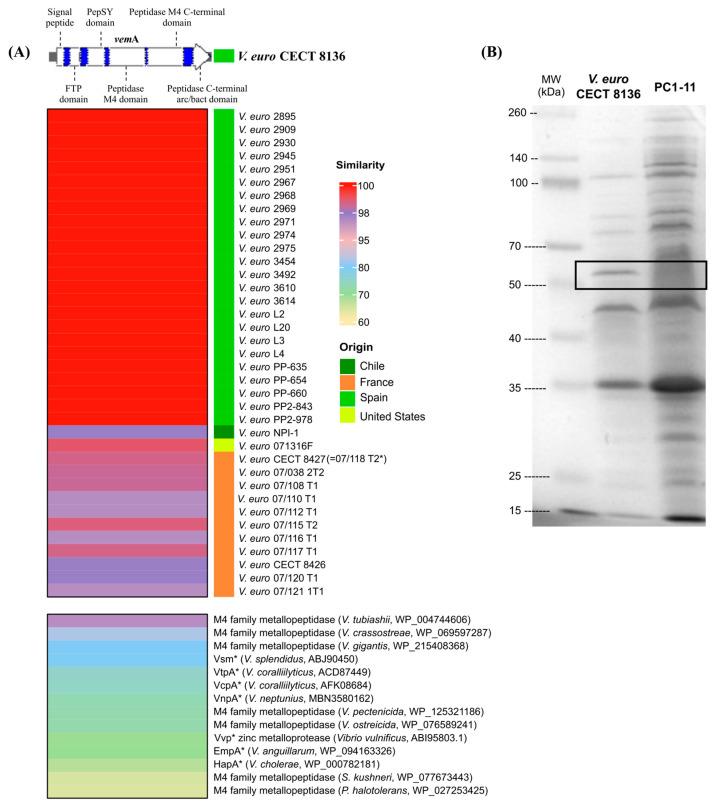
(**A**) Conserved domains and homology of the (**B**) extracellular VemA protein secreted by *V. europaeus* CECT8136. (**A**) Homology matches included were >60% and ranged between 63.95% (*Photobacterium halotolerans*) and 100% using the strain CECT8136 as reference. Asterisks indicate homologous genes in which its role in virulence was studied and published. (**B**) VemA protein is highlighted within a black square.

Locus WP_069668927 (1824 bp) from the *V. europaeus* CECT 8136 genome was designated as vemA (*V. europaeus* metalloprotease A) and encodes for VemA, a vibriolysin belonging to the M4 family of metallopeptidases and gluzincins subfamily [33]. VemA (608 aa; Figure 1A) showed the typical structure of vibriolysins [33], they are synthesized as a pro-peptide precursor containing a signal peptide signature in the N-terminal region (1–75 bp) and four conserved regions: (i) an FTP (fungalysin/thermolysin pro-peptide) domain (164–281 bp); (ii) a PepSY domain (349–569 bp); (iii) an M4 neutral protease which includes two M4 domains, such as peptidase M4 domain (622–1050 bp) and peptidase M4 C-terminal domain (1057–1490 bp); and (iv) a C-terminal pre-peptidase domain (1582–1775 bp). The mature forms of VemA contain the conservative region with M4 neutral proteases, which is also known as the catalytic domain [33]. Interestingly, VemA is classified as a vibriolysin-like protein within the clade II (VLP-II), which includes vibriolysins encoded by other opportunist pathogens, such as *V. coralliilyticus* [33].

**Figure 2:**
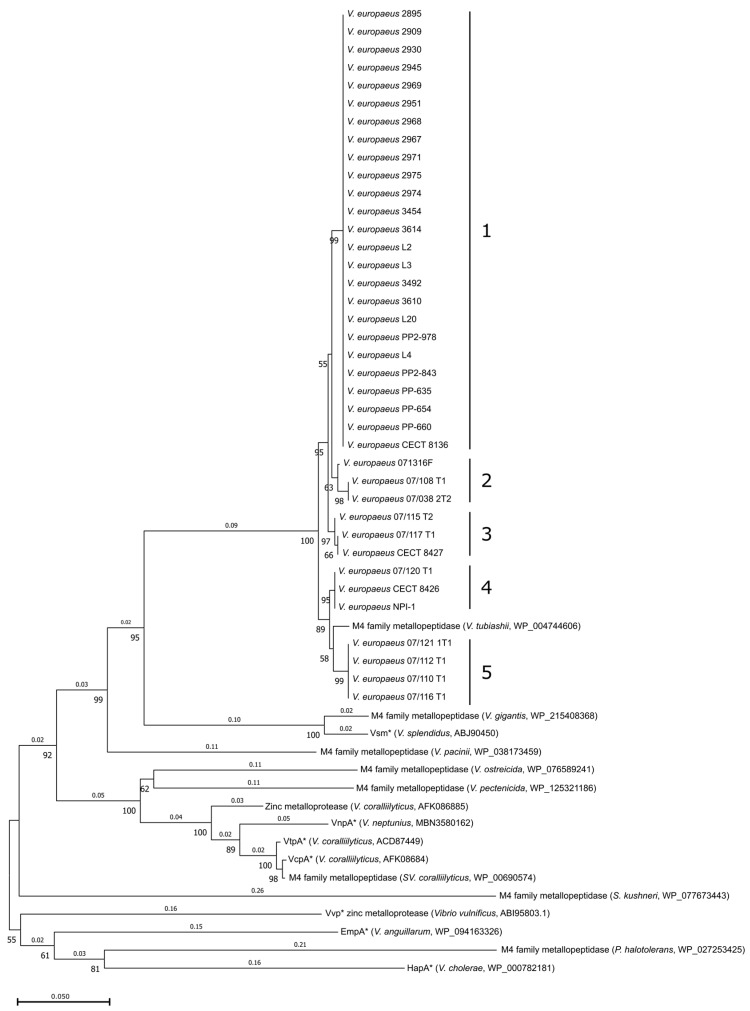
Phylogenetic tree of the VemA protein and its homologous proteins. Phylogenetic tree based on the homologous proteins obtained by the NJ method. Horizontal branch lengths are proportional to evolutionary divergence (showed on each branch). Bootstrap support (≥50%) from 1000 replicates appears next to the corresponding branch. Intraspecific clusters are designated as 1–5. Asterisks indicate homologous genes in which its role in virulence was studied and published.

VemA was encoded by all *V. europaeus* strains (n = 38 genomes/strains; Table S1) showing a high similarity (>97%) with the reference strain CECT8136 by BLASTp at the intraspecific level (Figure 1A). Phylogenetic analysis (Figure 2) indicated that Spanish isolates (n = 24 strains) constituted a monophyletic group (cluster #1; bootstrap value = 99; homology = 100%). However, the remaining strains were split among four additional clusters: cluster #2 (two French strains closely related with the American strain 07136F; bootstrap value = 63; homology = 99.01–98.52%); cluster #3 (French strains; bootstrap value = 97; homology = 98.85–98.68%); cluster #4 (two French strains, including the strain 07/118 T2 = CECT 8126 studied by Mersni-Achour et al. [13], were closely related with the Chilean strain NPI-1; bootstrap value = 95; homology = 97.85%); and cluster #5 (four French strains closely related with *V. tubiashii*; bootstrap value = 58; homology = 97.03%).

The homology search with other bacterial species revealed VemA as a homolog of other zinc-metalloproteases described in bivalve pathogens, such as *V. tubiashii*, *V. crassostreae*, *V. splendidus*, *V. coralliilyticus*, *V. neptunius*, *V. pectenicida* and *V. ostreicida* (Figure 1A and Figure 2; Table S3). In addition, some of those proteins have been reported as virulence factors in *V. splendidus* (Vsm = 79.57% similarity), *V. coralliilyticus* (VtpA = 75.16% and VcpA = 74.84% similarity), *V. neptunius* (VnpA = 72.95% similarity) or *V. cholerae* (HapA = 67.38% similarity).

### 3.2. VemA Is an Extracellular Protein Necessary for the Full Virulence of V. europaeus Although It Is Not a Major Virulence Factor

An in-frame deletion mutant, designated as PC1-11 (Table 1), was produced from *V. europaeus* CECT 8136 to study the role of the *vemA* gene in infection challenges using Manila clam juveniles and larvae. Interestingly, a polyacrylamide gel used to gain insight into the VemA protein and the analysis of the ECPs between the wildtype and PC1-11 showed a protein band of ~66 kDa corresponding to VemA (Figure 1B).

**Figure 3:**
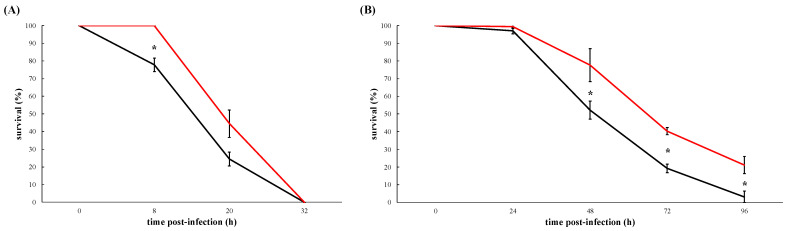
Survival rates after challenge Manila clam juveniles (**A**) and larvae (**B**) with *V. europaeus* CECT 8136 (black curves) and *ΔvemA* mutant (PC1-11, red curves). Values are a mean of three replicates and SD is displayed by error bars. Mortalities in negative controls inoculated with *V. breoganii* C5.5 were not detected in juveniles after 80 h (**A**) and below 5% for larvae (**B**) (data not shown). Asterisks indicate significant differences.

In juveniles, the infection due to the wildtype (*V. europaeus* CECT 8136) caused mortalities faster than the ΔvemA mutant (wildtype vs. PC1-11): 22% vs. 0% after 8 h (*p* = 0.034); 76% vs. 56% after 20 h (*p* = 0.44); and 100% in both cases after 32 h post-infection (Figure 3A).

Results from virulence challenges performed on larvae followed the same mortality dynamic than juveniles (Figure 3B). Larval mortalities due to the wildtype were ~20% higher than PC1-11 (wildtype vs. PC1-11): 48% vs. 22% after 48 h (*p* = 0.05); 81% vs. 60% after 72 h (*p* = 0.05) and 97% vs. 79% after 96 h (*p* = 0.05) post-infection (Figure 2B).

Our results showed that VemA is an extracellular protein and in absence of the *vemA* gene mass mortalities of larvae and juveniles are slowed down although finally those mortalities are unavoidable reaching high mortality rates: 100% for juveniles and 80% for larvae (Figure 2A,B). These results demonstrate that VemA is not a major virulence factor although in its presence *V. europaeus* kills larvae and juveniles faster, specially at the beginning of the infection.

### 3.3. V. europaeus Forms Biofilms on Manila Clam Mucus

**Figure 4:**
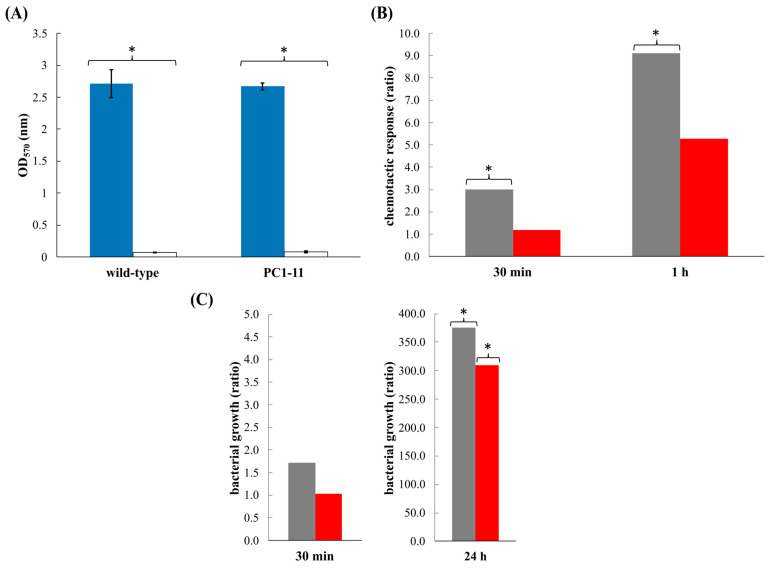
(**A**) Biofilm formation, (**B**) chemotaxis and (**C**) bacterial growth on body mucus by *V. europaeus* CECT 8136 ((**B**,**C**) grey columns) and PC1-11 mutant ((**B**,**C**) red columns). (**A**) Values are a mean of eight replicates and SD is displayed on the columns. Results showed the biofilm formation in the presence of mucus (blue columns) or in its absence (white columns). (**B**) The data shown are means of three independent replicates and the chemotactic responses are expressed as the ratio between the number of bacteria in mucus capillaries and the number of bacteria in control capillaries only in the presence of chemotaxis buffer after 30 min and 1 h. (**C**) The data shown are means of two independent replicates and the bacterial proliferation is expressed as the ratio between the number of bacteria in mucus after incubation and the number of bacteria at the beginning of the experiment (time 0). Asterisks indicate significant differences.

Body mucus (protein concentration = 2.3 μg mL^−1^) from Manila clams did not show any bactericidal effect on solid media in *V. europaeus* wildtype or PC1-1. Thus, we have evaluated the adherence by means of a bacterial biofilm formation over a surface impregnated with Manila clam mucus (Figure 4A). Interestingly, wildtype and PC1-11 exhibited a similar attachment pattern to bivalve mucus and to polystyrene, in both cases being significantly higher towards mucus than polystyrene (*p* = 0 for wildtype and PC1-11) (Figure 4A). These results demonstrate the ability of *V. europaeus* to form biofilms on bivalve mucus.

### 3.4. V. europaeus Is Attracted by Mucus and VemA Participates in Bacterial Chemotaxis

The wildtype strain was significantly attracted to bivalve mucus, approximately three times more after 30 min (*p* = 0.042) and ~9× after 1 h (*p* = 0.014) in relation to the control in the absence of mucus (Figure 4B). PC1-11 exhibited a decreased chemoattraction towards bivalve mucus after 30 min (*p* = 0.348) and 1 h (*p* = 0.122) in relation to the control (Figure 4B). Those results suggest that VemA plays a role in the host’s colonization attracted by the mucus.

### 3.5. VemA Promotes the Proliferation of V. europaeus over the Mucus Matrix

Wild-type and PC1-11 were able to grow over the mucus matrix (Figure 4C). As we observed previously by chemotaxis, the bacterial proliferation of PC1-11 was similar to the negative control after 30 min (*p* = 0.904) and only wildtype showed a ratio higher than 1 after 30 min (*p* = 0.517) (Figure 4C). Bacterial growth was significant higher (300×) for wildtype (*p* = 0.017) and PC1-11 (*p* = 0.004) than the negative controls after 24 h (Figure 4C). Although similarly, it is important to note that bacterial proliferation was slightly lower for PC1-11 (ratio = 309.3) than wildtype (ratio = 375.7).

## 4. Discussion

The role of M4 metalloproteases such as vibriolysins in *Vibrio* virulence is not clear [33]. For instance, pathogenic microorganisms, such as *V. cholerae*, *V. vulnificus*, *V. anguillarum* or *V. neptunius*, use vibriolysins as their main virulence factor [22,34,35,36]. Our results have demonstrated that *vemA* is a core gene highly conserved among all *V. europaeus* strains and even in other related species as the closest relative *V. tubiashii* [4,14]. Interestingly, VemA is a homolog of other zinc-metalloproteases encoded by non-pathogenic species, such as *V. gigantis* (Figure 1A), suggesting other functions not related to virulence [33]. In fact, VemA is classified as a vibriolysin-like protein belonging to clade II (VLP–II), which includes opportunist pathogens such as *V. coralliilyticus* and non-pathogenic species such *V. pacinii* [33].

The first part of this study was to elucidate the contribution degree of VemA as a major virulence factor (or not) in the virulence of *V. europaeus* towards Manila clam juveniles and larvae. Previous results described VemA as the major toxicity factor when ECPs secreted by *V. europaeus* 07/118 T2 (=CECT 8126; Figure 1A) were inoculated with Pacific oyster larvae, making VemA responsible of the 70% mortality (other proteins were required to obtain a full virulence) [13]. However, we have demonstrated in in vivo challenges that VemA is not a major virulence factor when live cells (Δ*vemA* and wildtype) are used instead ECPs because mass mortalities were unavoidable in all cases. 

Divergences between the results obtained when live cells (mutants and wildtype) were used rather than ECPs in in vivo experimental challenges were also described for other metalloproteases homologous to VemA. For instance, Vsm, secreted by the bivalve pathogen *V. splendidus*, was described as the major toxicity factor by comparisons between the ECPs secreted by the defective mutant Δ*vsm* and the wildtype. In contrast, when a live mutant (Δ*vsm*) was injected into oysters the mortality rates were similar to the wildtype, promoting mass mortalities in both cases [23,37]. Binese et al. [37] found differential expression of different proteins detected in wildtype and Δ*vsm* mutant ECPs: the Δ*vsm* secreted a predicted protein of unknown function (VS2864) and an additional metalloprotease of the M6 family (VSA1062) while wildtype ECPs contained an unknown protein (VSA576) and three spots corresponding to Vsm. Same divergences were reported for other bivalve pathogens, such as *V. coralliilyticus.* First, Sussman et al. [20] identified a zinc-metalloprotease (VcpA) secreted by *V. coralliilyticus* P1 and demonstrated its key role in virulence by testing ECPs from mutants (Δ*vcpA*) vs. wildtype. However, the VcpA contribution to *V. coralliilyticus* virulence was different when live cells were used, and similar high-mortality rates were detected due to wildtype and the mutants (Δ*vcpA*) [38]. In this case, the Δ*vcpA* mutant showed a higher haemolytic activity and secreted 18 proteins not secreted by the wildtype, including four types of metalloproteases, a chitinase, a haemolysing-related protein RbmC, the Hcp protein and 12 hypothetical proteins [38]. Altogether, these studies indicate a diverse virulence repertoire that possibly enables both *Vibrio* species to be efficient animal pathogens [37,38]. Our hypothesis is that other additional factors may have a significant contribution to the degree of virulence of *V. europaeus* that might compensate for some of the functions fulfilled by the product of the deleted *vemA* gene. Thus, the deletion of a virulence factor, such as a VemA, can be compensated for by the upregulation of other proteins by live bacteria in an in vivo challenge. This must be addressed by proteomic analyses in further studies.

The second part of this study was to elucidate the role of VemA in virulence. A dramatic characteristic of vibriosis due to pathogenic *Vibrio* spp., including *V. europaeus*, is rapid proliferation of the bacterial pathogen inside the animal, and thus its sudden and fatal massive mortalities in bivalve aquacultures [4,39]. This negatively limits the application and efficacy of specific treatments once disease has been detected [4,39]. Our results showed that the infection process was faster when Manila clam larvae and juveniles were infected with the wildtype rather than the Δ*vemA* mutant. Despite VemA not being a major virulence factor, it plays a role in virulence at the beginning of the infection and thus affecting the “sudden and fatal” feature of vibriosis. To address this, we focused on the mucus because this is the first line of defence against microorganisms [2]. Different metalloproteases participate in chemotaxis such as the metalloprotease Vvp secreted by *V. vulnificus* towards eel mucus [32]. Interestingly the transcription of the metalloprotease EmpA by the fish pathogen *V. anguillarum* was induced by mucus [40]. In *V. cholerae* the haemagglutinin protease HapA is an important virulence factor attributed to multiple pathogenic activities, including degradation of mucus barriers in human intestines [41,42,43]. In *V. coralliilyticus* the zinc-metalloprotease VcpB, that causes photoinactivation of coral endosymbionts and coral tissue lesions, was one of the most significantly and strongly upregulated genes in coral mucus at 10 and 60 min [44]. Our results revealed that *V. europaeus* was able to resist the bactericidal action of mucus and display a chemotaxis ability to colonize the body mucus of clams by forming biofilms. Interestingly, it has been reported that vibriolysins have a synergistic effect with collagenase or haemagglutinin in tissue degradation [45,46]. In fact, VemA contains a C-terminal pro-peptide domain which can help protease to cohere substrate causing the enhancement of hydrolysis efficiency [47,48]. In conclusion, the overall results suggest that VemA, although it is not a major virulence factor, plays a role in the colonization of the Manila clam mucus, and thus boosts the infection process as we observed in virulence challenge experiments.

## Figures and Tables

**Table 1:** List of plasmids and bacterial strains used in this study.

Bacterial Strains and Plasmids	Description	Reference
CECT 8136	*V. europaeus* wildtype strain	Dubert et al. [14]
PC1-11	CECT 8136 Δ*vemA*	This study
C5.5	*Vibrio breoganii*	Beaz-Hidalgo et al. [24]
*E. coli* Π3813	B462 Δ*thyA*::(erm-pir-116) (Ery^R^)	Le Roux et al. [23]
*E. coli* β3914	β2163 gyrA462 zei-298::Tn10 (Kn^R^ Ery^R^)	Le Roux et al. [23]
pSW7848T	pSW23T::*araC*-P_BAD_*ccdB* (Cm^R^)	Val et al. [25]
pPC1	pSW7848T flanked by two homologous regions upstream/downstream *vemA*	This study

## Data Availability

Not applicable.

## Supplementary Materials

The following supporting information can be downloaded at: https://www.mdpi.com/article/10.3390/microorganisms10122475/s1, Table S1. *V. europaeus* strains/genomes used in this study; Table S2. Primers used in this study; Table S3. Identities (%) obtained from interspecific homology search; Figure S1. Map of the recombinant plasmid pPC1.
