# Supplementary material for: Role of the Vibriolysin VemA Secreted by the Emergent Pathogen Vibrio europaeus in the Colonization of Manila Clam Mucus
Source: Microorganisms. 2022 Dec 15;10(12):2475. doi: 10.3390/microorganisms10122475 (PMC9785129; doi:10.3390/microorganisms10122475)
Supplement: Supplementary file 1 [file microorganisms-10-02475-s001.zip › microorganisms-2055147-supplementary.pdf]

Table S1. *V. europaeus* strains/genomes used in this study.

Strain:	Source:	Isolation mortalities:	Location:	Date:	Reference:	Assembly
PP-654	Flat oyster larvae (*Ostrea edulis*)	Y	Hatchery CIMA (Ribadeo; Galicia, Spain)	March/2001	Prado et al. [9]	-
PP-660	Flat oyster larvae (*Ostrea edulis*)	Y	Hatchery CIMA (Ribadeo; Galicia, Spain)	March/2001	Prado et al. [9]	-
PP-635	Seawater tank; Flat oyster larvae (*Ostrea edulis*)	Y	Hatchery CIMA (Ribadeo; Galicia, Spain)	March/2001	Prado et al. [9]	-
CECT 8136 (PP-638)	Seawater tank; Flat oyster larvae (*Ostrea edulis*)	Y	Hatchery CIMA (Ribadeo; Galicia, Spain)	March/2001	Saulnier et al. [7] / Travers et al. [8]	GCA_001695575.1
04/002 1T2 (CECT 8427)	Abalone spat (*Haliotis tuberculata*)	Y	Hatchery (Cotentin; Normandy, France)	January/2004	Saulnier et al. [7] / Travers et al. [8]	-
07/118 T2 (CECT 8426)	Pacific oyster spat (*Crassostrea gigas*)	Y	Hatchery (La Tremblade; Nouvelle-Aquitaine, France)	June/2007	Saulnier et al. [7] / Travers et al. [8]	GCA_015654285.1
07/038 2T2	Pacific oyster spat (*Crassostrea gigas*)	Y	Hatchery (La Tremblade; Nouvelle-Aquitaine, France)	August/2007	Saulnier et al. [7] / Travers et al. [8]	-
07/108 T1	Pacific oyster spat (*Crassostrea gigas*)	Y	Hatchery (La Tremblade; Nouvelle-Aquitaine, France)	August/2007	Travers et al. [8]	-
07/110 T1	Pacific oyster spat (*Crassostrea gigas*)	Y	Hatchery (La Tremblade; Nouvelle-Aquitaine, France)	August/2007	Saulnier et al. [7] / Travers et al. [8]	-
07/112 T1	Pacific oyster spat (*Crassostrea gigas*)	Y	Hatchery (La Tremblade; Nouvelle-Aquitaine, France)	August/2007	Travers et al. [8]	-
07/115 T2	?	?	?	?/2007	-	-
07/116 T1	Pacific oyster spat (*Crassostrea gigas*)	Y	Hatchery (La Tremblade; Nouvelle-Aquitaine, France)	August/2007	Travers et al. [8]	-
07/117 T1	Pacific oyster spat (*Crassostrea gigas*)	Y	Hatchery (La Tremblade; Nouvelle-Aquitaine, France)	August/2007	Saulnier et al. [7] / Travers et al. [8]	-
07/120 T1	Pacific oyster spat (*Crassostrea gigas*)	Y	Hatchery (La Tremblade; Nouvelle-Aquitaine, France)	August/2007	Travers et al. [8]	-
07/121 1T1	?	?	?	?/2007	-	-
PP2-843	Manila clam spat (early spat ≥1000 µm) (*Ruditapes philippinarum*)	Y	Hatchery Ostreira (Barizo; Galicia, Spain)	November/2008	Prado et al. [9]	-
PP2-978	Manila clam spat (early spat ≥1000 µm) (*Ruditapes philippinarum*)	Y	Hatchery Ostreira (Barizo; Galicia, Spain)	November/2008	Prado et al. [9]	-
2909	Seawater tank; Carpet shell clam larvae (*Ruditapes decussatus*)	Y	Hatchery CIMA (Ribadeo; Galicia, Spain)	May/2011	Dubert et al. [10]	-
2895	Seawater tank; Carpet shell clam larvae (*Ruditapes decussatus*)	Y	Hatchery CIMA (Ribadeo; Galicia, Spain)	May/2011	Dubert et al. [10]	-
2930	Carpet shell clam larvae (*Ruditapes decussatus*)	Y	Hatchery CIMA (Ribadeo; Galicia, Spain)	May/2011	Dubert et al. [10]	-
2951	Carpet shell clam larvae (*Ruditapes decussatus*)	Y	Hatchery CIMA (Ribadeo; Galicia, Spain)	May/2011	Dubert et al. [10]	-
2945	Seawater tank; Carpet shell clam larvae (*Ruditapes decussatus*)	Y	Hatchery CIMA (Ribadeo; Galicia, Spain)	May/2011	Dubert et al. [10]	-
2967	Carpet shell clam larvae (*Ruditapes decussatus*)	Y	Hatchery CIMA (Ribadeo; Galicia, Spain)	May/2011	Dubert et al. [10]	-
2968	Seawater tank; Carpet shell clam larvae (*Ruditapes decussatus*)	Y	Hatchery CIMA (Ribadeo; Galicia, Spain)	May/2011	Dubert et al. [10]	-
2969	Carpet shell clam larvae (*Ruditapes decussatus*)	Y	Hatchery CIMA (Ribadeo; Galicia, Spain)	May/2011	Dubert et al. [10]	-
2971	Seawater tank; Carpet shell clam larvae (*Ruditapes decussatus*)	Y	Hatchery CIMA (Ribadeo; Galicia, Spain)	May/2011	Dubert et al. [10]	-

2974	Carpet shell clam larvae (*Ruditapes decussatus*)	Y	Hatchery CIMA (Ribadeo; Galicia, Spain)	May/2011	Dubert et al. [10]	-
2975	Seawater tank; Carpet shell clam larvae (*Ruditapes decussatus*)	Y	Hatchery CIMA (Ribadeo; Galicia, Spain)	May/2011	Dubert et al. [10]	-
3454	Wedge clam larvae (*Donax trunculus*)	Y	Hatchery CIMA (Ribadeo; Galicia, Spain)	May/2012	-	-
3492	Seawater tank; Wedge clam larvae (*Donax trunculus*)	Y	Hatchery CIMA (Ribadeo; Galicia, Spain)	June/2012	-	-
3610	Carpet shell clam broodstock (*Ruditapes decussatus*)	N	Hatchery CIMA (Ribadeo; Galicia, Spain)	July/2012	Dubert et al. [16]	-
3614	Carpet shell clam eggs (*Ruditapes decussatus*)	N	Hatchery CIMA (Ribadeo; Galicia, Spain)	July/2012	Dubert et al. [16]	-
NPI1	Peruvian scallop larvae (*Argopecten purpuratus*)	Y	Hatchery Invertec Ostimar (Coquimbo, Chile)	February/2015	Rojas et al. [11]	GCA_013154935.1
071316F	Seawater	N	Netarts Bay (Oregon, US)	July/2016	Rojas et al. [11]	GCA_013114045.1
L2	Banded carpet shell larvae (*Polititapes rhomboides*)	Y	Hatchery CIMA (Ribadeo; Galicia, Spain)	March/2018	-	-
L3	Razor shell clam larvae (*Ensis arcuatus*)	Y	Hatchery CIMA (Ribadeo; Galicia, Spain)	March/2018	-	-
L4	Razor shell clam larvae (*Ensis arcuatus*)	Y	Hatchery CIMA (Ribadeo; Galicia, Spain)	March/2018	-	-
L20	Manila clam larvae (*Ruditapes philippinarum*)	Y	Hatchery CIMA (Ribadeo; Galicia, Spain)	May/2018	-	-
EX1	Flat oyster larvae (*Ostrea edulis*)	Y	Hatchery Ostreira (Arousa; Galicia, Spain)	July/1985	Lodeiros et al. [15]	-

Table S2. Primers used in this study.

**Use**	**Name**	**Sequence 5'-3'**	**Reference**
Construction of recombinant plasmid pPC1:	F1_PC1_f	agcttgatatcgaattcctgcagcaatcgttagtttagacag	This study
	F1_PC1_r	tcaagctttagtgtcacgccagattggttcattattctttcctg	This study
	F2_PC1_f	ggagtttctacaaataacaggaaagaataatgaaccaatctggcgtgacactaaag	This study
	F2_PC1_r	cataattggtaacgaatcagacaattcaaaccttcaagtaactgctg	This study
	pSW_PC1_for	cagcagttacttgaaggtttgaattgtctgattcgttaccaattatg	This study
	pSW_PC1_rev	ctgtctaaactaacgattgctgcaggaattcgatatcaagc	This study
Primers for plasmid sequencing:	seq_F1_PC1_f	gcaatcgttagtttagaca	This study
	seq_F1_PC1_r	ttggttcattattctttcctg	This study
	seq_F2_PC1_f	tctggcgtgacactaaag	This study
	seq_F2_PC1_r	caaaccttcaagtaactgctg	This study
Verification of the first allelic exchange after conjugation:	Check1_ins_pSW_for	cgggcaggataggtgaagtag	Hussain et al. [29]
	Check1_ins_pSW_rev	gagtttcgtgccggttgtg	Hussain et al. [29]
Verification of the second allelic exchange:	check_mut_PC1_f	tgcgaacccacctaagatagc	This study
	check_mut_PC1_r	agaggccaatcggtaacagg	This study

Table S3. Identities (%) obtained from interspecific homology search.

**Protein**	**Specie**	**No. Accesion**	**Identity (%)**
M4 family metallopeptidase	*V. tubiashii*	WP_004744606	97.04
M4 family metallopeptidase	*V. crassostreae*	WP_069597287	83.76
M4 family metallopeptidase	*V. gigantis*	WP_215408368	79.74
Vsm*	*V. splendidus*	ABJ90450	79.57
VtpA*	*V. coralliilyticus*	ACD87449	75.16
VcpA*	*V. coralliilyticus*	AFK08684	74.84
VnpA*	*V. neptunius*	MBN3580162	72.95
M4 family metallopeptidase	*V. pectenicida*	WP_125321186	72.46
M4 family metallopeptidase	*V. ostreicida*	WP_076589241	72.37
EmpA*	*V. anguillarum*	WP_094163326	69.74
HapA*	*V. cholerae*	WP_000782181	67.38
M4 family metallopeptidase	*S. kushneri*	WP_077673443	64.43
M4 family metallopeptidase	*P. halotolerans*	WP_027253425	63.95

Asterisks indicate homologous genes in which its role in virulence was studied and published.

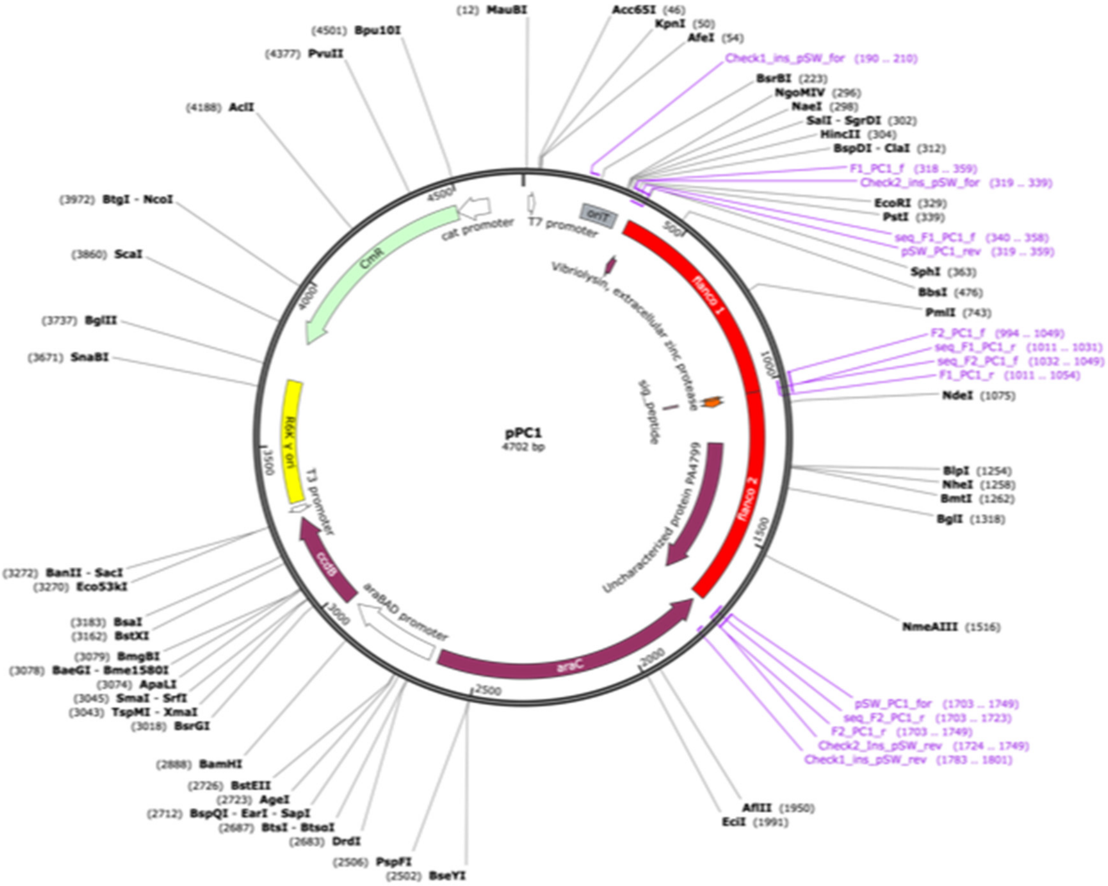

Figure S1. Map of the recombinant plasmid pPC1.

Primers (described on Table S2) and restriction enzymes (unique cut) are highlighted on the plasmid.
